# Sea ice meiofauna distribution on local to pan‐Arctic scales

**DOI:** 10.1002/ece3.3797

**Published:** 2018-01-29

**Authors:** Bodil A. Bluhm, Haakon Hop, Mikko Vihtakari, Rolf Gradinger, Katrin Iken, Igor A. Melnikov, Janne E. Søreide

**Affiliations:** ^1^ Department of Arctic and Marine Biology Faculty of Biosciences, Fisheries and Economics UiT The Arctic University of Norway Tromsø Norway; ^2^ College of Fisheries and Ocean Sciences University of Alaska Fairbanks Fairbanks AK USA; ^3^ Norwegian Polar Institute, Fram Centre Tromsø Norway; ^4^ Shirshov Institute of Oceanology Russian Academy of Sciences Moscow Russia; ^5^ The University Centre in Svalbard Longyearbyen Norway

**Keywords:** Arctic, biodiversity, environmental monitoring, meiofauna, sea ice, spatial and temporal scales

## Abstract

Arctic sea ice provides microhabitats for biota that inhabit the liquid‐filled network of brine channels and the ice–water interface. We used meta‐analysis of 23 published and unpublished datasets comprising 721 ice cores to synthesize the variability in composition and abundance of sea ice meiofauna at spatial scales ranging from within a single ice core to pan‐Arctic and seasonal scales. Two‐thirds of meiofauna individuals occurred in the bottom 10 cm of the ice. Locally, replicate cores taken within meters of each other were broadly similar in meiofauna composition and abundance, while those a few km apart varied more; 75% of variation was explained by station. At the regional scale (Bering Sea first‐year ice), meiofauna abundance varied over two orders of magnitude. At the pan‐Arctic scale, the same phyla were found across the region, with taxa that have resting stages or tolerance to extreme conditions (e.g., nematodes and rotifers) dominating abundances. Meroplankton, however, was restricted to nearshore locations and landfast sea ice. Light availability, ice thickness, and distance from land were significant predictor variables for community composition on different scales. On a seasonal scale, abundances varied broadly for all taxa and in relation to the annual ice algal bloom cycle in both landfast and pack ice. Documentation of ice biota composition, abundance, and natural variability is critical for evaluating responses to decline in Arctic sea ice. Consistent methodology and protocols must be established for comparability of meiofauna monitoring across the Arctic. We recommend to (1) increase taxonomic resolution of sea ice meiofauna, (2) focus sampling on times of peak abundance when seasonal sampling is impossible, (3) include the bottom 30 cm of ice cores rather than only bottom 10 cm, (4) preserve specimens for molecular analysis to improve taxonomic resolution, and (5) formulate a trait‐based framework that relates to ecosystem functioning.

## 1 INTRODUCTION

**Figure 1:**
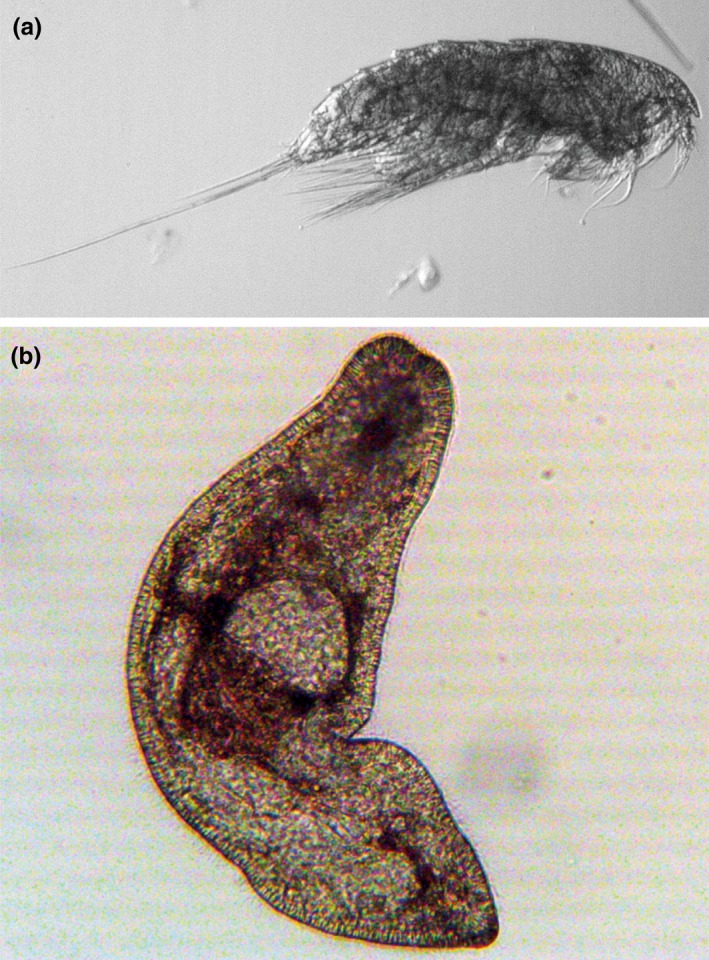
Common meiofaunal taxa associated with sea ice include (a) harpacticoid copepods and (b) flatworms. Photographs: (a) Julia Ehrlich, Alfred Wegener Institute for Polar and Marine Research, (b) Kyle Dilliplaine, University of Alaska Fairbanks

Arctic sea ice provides a wide range of microhabitats for biota that inhabits the liquid‐filled network of brine channels and the ice–water interface (Hunt et al., 2016). Among the multicellular organisms, sea ice meiofauna (= sympagic meiofauna) is arguably the most poorly studied regarding their diversity, abundance, variability in time and space, and ecological role in the sea ice system. Sympagic meiofauna comprises multicellular organisms such as nematodes, harpacticoid copepods, flatworms, and rotifers (Figure 1), typically ranging from ~20 to 500 μm in size. Single‐celled ciliates are also included as meiofauna in some studies, but in others, these are referred to as microfauna (<62 μm; Carey, 1985; Bluhm, Swadling, & Gradinger, 2017). In addition to ice‐endemic species, both pelagic and benthic meiofaunal species occur in sea ice, often as larvae or juvenile stages. Meiofaunal species or stages settle in sea ice through active migration, are picked up from the water column during ice formation, disperse from multiyear ice, or are recruited from resting stages (Carey & Montagna, 1982). Besides adding to biodiversity of the Arctic, the role of many ice meiofauna taxa relates to their grazing on the seasonally abundant and highly concentrated ice algae (Grainger & Hsiao, 1990) that allows for high meiofaunal growth rates early in the season before the phytoplankton bloom develops (McConnell, Gradinger, Iken, & Bluhm, 2012). Yet, meiofauna grazing does not appear to limit ice algal growth despite their seasonally high abundances, often with >200,000 meiofaunal individuals/m^2^ in landfast ice at peak times (Bluhm, Hop, et al., 2017; Gradinger, Kaufman, & Bluhm, 2009). The ingestion rates by multicellular meiofauna are estimated at <10% of ice algal biomass (Gradinger, Friedrich, & Spindler, 1999; Michel, Nielsen, Nozais, & Gosselin, 2002). Such low grazing pressure may in part be related to the size of the brine channel system, mostly <1 mm in a given channel of solid ice, which may restrict some meiofauna from exploiting niches with high ice algal growth (Krembs, Gradinger, & Spindler, 2000). This restriction, however, rapidly changes during the onset of melt when the porosity increases above the percolation threshold of about 5% brine volume fraction (Golden, Ackley, & Lytle, 1998) and the brine channels connect so that organisms in the ice can move about. Direct absorption of ice‐produced dissolved organic matter has been suggested as an alternative feeding mode for sympagic nematodes (Tchesunov & Riemann, 1995). Meiofaunal predators, in turn, appear to be rare (Bluhm, Swadling, et al., 2017), perhaps due to space limitations within the brine channels and prey patchiness. Exploiting the sea ice as nursery ground can, therefore, be a winning life strategy for those organisms capable of living in this extreme environment due to the high concentrations of food and low predation pressure. During melt, ice‐derived organic matter including ice meiofauna is released from the sea ice and provides food for pelagic and benthic biota (Moran et al., 2012).

Sea ice extent in the Arctic Ocean has declined by over 30% since the satellite record began in 1979 (Meier et al., 2014; Vaughan et al., 2013), and the reduction in sea ice has occurred during all months of the year (Barber et al., 2015). The average age of individual Arctic ice floes has decreased from multiyear to mainly first‐ and second‐year sea ice, generally decreasing the thickness of Arctic sea ice with about 0.5 m from the 1980s to 2010s (Bi et al., 2016; Perovich et al., 2015). Multiyear sea ice, which used to cover about 75% of the Arctic in 1983, is currently limited to the areas north of Greenland, the central Arctic, and parts of the Canadian Arctic Archipelago with as little as 62% of the summer ice cover remaining compared to 1978–1988 values (Maslanik et al., 2007; Meier et al., 2014; Stroeve et al., 2012). First‐year ice has concomitantly increased in relative proportion and importance, although dates of its freeze‐up and breakup have also shifted substantially (Markus, Stroeve, & Miller, 2009), leaving large parts of the Arctic without sea ice for an increasing amount of time (Arrigo, Matrai, & van Dijken, 2011). Consequences for sea ice biota—from bacteria to polar bears—seem inevitable, but are largely undocumented (CAFF, 2017). Documentation of ice biota composition, abundance, and natural variability is critical for evaluating responses to the decline in Arctic sea ice.

Although sea ice meiofauna has been known to occur for over a century (Nansen, 1906), and its biodiversity has been documented at various levels (Bluhm, Hop, et al., 2017; Bluhm, Swadling, et al., 2017; Poulin et al., 2011), a quantitative pan‐Arctic synthesis of sea ice meiofauna composition and abundance has been lacking to date. Distribution and composition of sea ice meiofauna have been reported from some locations in the Arctic, for example, the Canada Basin (Gradinger, Bluhm, & Iken, 2010), Beaufort Sea (Marquardt, Kramer, Carnat, & Werner, 2011), Frobisher Bay in Arctic Canada (Grainger, Mohammed, & Lovrity, 1985; Nozais, Gosselin, Michel, & Tita, 2001), Hudson Bay (Grainger, 1988), and Fram Strait (Schünemann & Werner, 2005). These studies documented both similar community patterns of ice meiofauna, such as likeness of taxa found at the class and phylum levels and concentration at near‐bottom layers of the sea ice, and different levels of abundance and dominant taxa. The identified patterns and variations have locally primarily been related to different ice types (landfast, first‐year, and multiyear ice), seasons, hydrographic conditions, snow cover, and sediment load (see Bluhm, Hop, et al., 2017 for review). Analyses at multiple spatial scales are still missing, but are needed in light of pressing questions related to biological consequences of sea ice decline.

In the State of the Arctic Biodiversity Report (CAFF, 2017), the Sea Ice Biota Expert Network of the Circumpolar Biodiversity Monitoring Programme (CBMP) of the Conservation of the Arctic Flora and Fauna (CAFF) assembled published and unpublished data sources of various ice biota occurrences throughout the Arctic. This study builds on the work on sea ice biota summarized in the report, but presents more extensive analyses of the underlying datasets related to sea ice meiofauna. Specifically, we ask here how variable taxonomic (phylum‐level) composition and abundance of sea ice meiofauna are at multiple spatial scales ranging from the vertical distribution within a single ice core at a given location—to the pan‐Arctic scale, and at a seasonal scale under landfast ice and pack ice conditions. Finally, we address management concerns with regard to challenges and requirements for monitoring sea ice biota, as well as future prospects for sea ice biota considering the dwindling Arctic sea ice cover.

## 2 METHODS

### 2.1 Data sources and spatial and temporal scales

**Figure 2:**
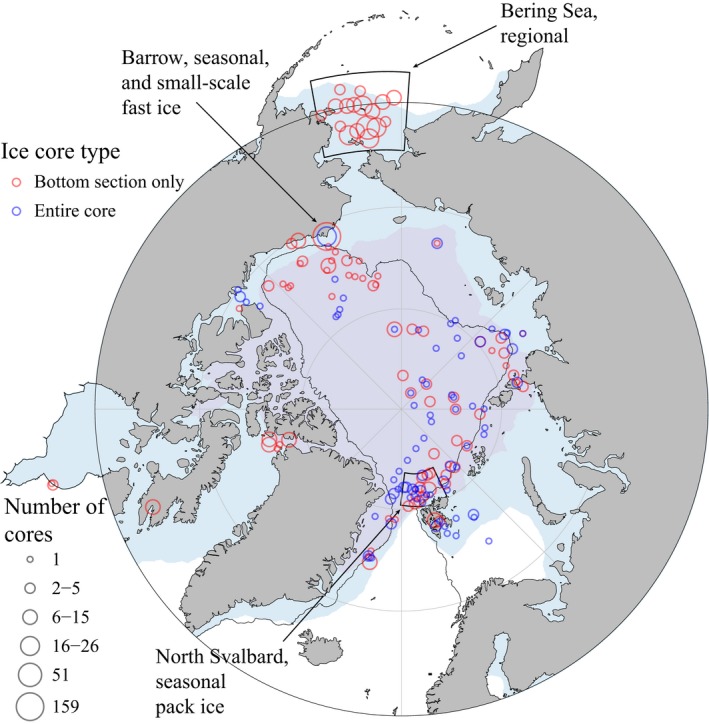
Pan‐Arctic map showing study locations sampled between 1979 and 2015, number of ice cores binned using 2° resolution, and median sea ice cover in March (blue fill) and September (purple fill) between 1981 and 2010 (when the majority of studies considered were conducted). The solid line separates the Arctic Ocean basins and shelf seas approximately following the 500 m isobath

For this study, we synthesized sea ice meiofauna data from literature and performed a meta‐analysis of these datasets combined with unpublished sea ice meiofauna information (sources listed in Table 1, locations in Figure 2, unpublished sources in Appendix S1). We only used data based on samples from ice cores and including at least information on abundance and taxonomic composition. We chose abundance over biomass data as biomass is rarely determined for sympagic meiofauna. Ice core sampling and processing are described in the sources listed in Table 1; previously unpublished data were mostly processed as described in Gradinger (2009) and Gradinger and Bluhm (2009), although with variable thicknesses of ice core sections. The resulting pan‐Arctic dataset was synthesized from 23 studies conducted from 1979 to 2015, with the majority conducted after 2000, and contained meiofauna composition and abundances from 721 ice cores. To address the question of spatial and temporal variability, the integrated dataset was used for analysis at four spatial scales and one temporal scale: (1) vertical distribution of sea ice meiofauna abundance within individual ice floes (i.e., ice cores). (2) Small‐scale spatial variability of abundance and taxonomic composition within a 50 km^2^ area sampled within 4 days. (3) Regional‐scale spatial variability, with ice meiofauna abundance and composition from the Bering Sea as an example. (4) Pan‐Arctic variability, to identify environmental factors contributing to variability in meiofauna abundance in general. (5) Seasonal variability at a landfast ice and a pack ice location.

**Table 1:** Studies included in the pan‐Arctic synthesis. Geographic regions for sampling (see Figure 6); total number of ice cores (*N*_total_); number of entire ice cores used for Figure 3 (*N*_whole_); abundance unit used in the study, defines the transformation equation used to calculate ind./m^2^ values (Appendix S2); sampling months and years; average distance from land (km) over all ice cores in a study; and reference, with further specifications of unpublished data sources in Appendix 1

Region	*N*_total_	*N*_whole_	Unit	Month	Year	Dist	Reference
Baffin Bay	23		Ind./m^2^	4, 5	1998, 1999	38.9	Nozais et al. (2001)
Barrow	171	20	Ind.	1–6 12	2005–2007	1.4	Bluhm and Gradinger^a^
Barrow	8		Ind./m^2^	2, 4, 5	2002, 2003	1.9	Gradinger et al. (2009)^b^
Beaufort Sea	2		Ind./m^2^	3, 5	1979	14.9	Carey and Montagna (1982)
Beaufort Sea	7		Ind./m^2^	4–6	1980	5.8	Kern and Carey (1983)
Beaufort Sea	6	5	Ind./L	4, 5	2008	32.7	Marquardt et al. (2011)
Bering Sea	236		Ind.	3–5	2008–2010	119.9	Gradinger, Iken & Bluhm, a^a^
Canada Basin	10	5	Ind./L	8, 9	2002, 2003	524.4	Gradinger et al. (2005)
Canada Basin	14		Ind./L	6, 7	2005	345.6	Gradinger et al. (2010)
Canada Basin	10		Ind./L	10	2009	414.5	Gradinger, Iken & Bluhm, b^a^
Central Basin, Eurasian Basin, Nansen Basin	39	5	Ind.	8, 9	2007	452.3	Kiko, Kern, Kramer, and Mütze (2017)^c^
Central Basin, Greenland Sea	8	8	Ind./L	8, 9	2001	477.1	Schünemann (2004)
Central Basin, Makarov Basin, Nansen Basin, Siberian Shelf Seas	11		Ind./m^2^	7, 8	1996	362.4	Friedrich^a^
Eurasian Basin, Greenland Sea, North Svalbard, Siberian Shelf Seas	57	3	Ind./L	7–9	1993, 1994	201.2	Friedrich (1997)^c^,^d^
Eurasian Basin, Greenland Sea, North Svalbard, South Svalbard	21		Ind./m^2^	5–7	1997	164.6	Werner & Friedrich^a^
Eurasian Basin, Greenland Sea, North Svalbard, South Svalbard	11	11	Ind./L	3, 4, 9	2002, 2003	140.3	Schünemann and Werner (2005)
Eurasian Basin, North Svalbard	39	3	Ind.	1, 3–6	2015	166.6	Hop^a^
Hudson Complex	8		Ind./m^2^	2, 3, 5, 6	1981, 1982	1.6	Grainger et al. (1985)
Hudson Complex	3		Ind./L	4	1983	4.9	Grainger (1988)
Makarov Basin	12		Ind_theo_	8, 9	2000	802.0	Melnikov, a^a^
Siberian Shelf Seas	4		Ind_theo_	4	2003	350.2	Melnikov, b^a^
South Svalbard	12		Ind.	4, 5	2009	2.8	Kramer^a^
South Svalbard	9	4	Ind.	5	2014	9.0	Søreide^a^
Total	721			1–10, 12	1979–1983, 1993, 1994, 1996–2003, 2005–2010, 2014, 2015	143.6	

a Unpublished data. See Appendix S1 for details about these data sources.

b Includes data from Gradinger and Bluhm (2005).

c Published partly in Kramer and Kiko (2011), but also includes previously unpublished data.

d Rotifera abundances from Friedrich and Smet (2000).

1. Variability within ice cores was determined by analyzing the vertical distribution of meiofauna abundance in 64 cores from studies that reported results for sectioned whole cores (Table 1). The ice core sections were divided into three categories. The first comprises the bottom sections of consistent length (0–10 cm) at the ice–water interface. The rest of the ice core was divided into two sections, top and middle. The top section was the uppermost ice section at the ice–snow interface; it varied in length between 6 and 59 cm, and top section cores were located between 40 and 348 cm from the ice–water interface. The remaining part of the ice core was called middle section and varied in length between 30 and 318 cm with cores located 10–328 cm from ice–water interface. Abundances within ice core sections were integrated to acquire the aforementioned grouping. Abundances within each ice core were transformed to percentage taxa inhabiting each section, and these percentages were further used to calculate aggregate percentages (see Abundance metrics section) for each of the categories by taxonomic group, calculated with bootstrapped 95% confidence intervals for mean estimates.
2. Meiofauna variability within small spatial scales of meters to kilometers was examined using data from a multiyear study in landfast ice off Barrow (recently renamed to its original native name Utqiaġvik), Alaska (Table 1). The bottom 10 cm of 29 ice cores sampled within 4 days in April 2007 were included in the analysis. Three to eleven replicate cores were taken at each of seven sites that were 3.2–8.6 km apart and all within a 50 km^2^ area. Absolute abundance and taxonomic group contribution were calculated for each individual core.
3. At the regional scale of tens to hundreds of kilometers, data from 59 sites within the central and northern Bering Sea (Figure 2), sampled between March and May 2008 and 2010, were analyzed for their ice meiofaunal taxonomic composition and abundance (Table 1). Three to nine replicate cores were sampled per site for 236 cores in total. Sampled ice cores were not sectioned consistently, which lead to the bottom 0–2 to 0–30 cm being used in the analyses, with corrections for different volumes (Appendix S2). Ice thickness varied between 19 and 152 cm. Mean abundance ($\overset{¯}{x}$), relative standard error ($\text{RSE}=100\%\times SE/\overset{¯}{x}$), and taxonomic group contribution were calculated for a 1° latitude × 2° longitude grid.
4. The entire synthesized dataset was used to examine meiofauna composition and abundance patterns on the pan‐Arctic scale (Figure 2). The Arctic region was categorized into 14 contiguous regions with common geographic, bathymetric, oceanographic, and sea ice features, and in several cases coherent sample distribution. This categorization gave a more detailed impression of ice meiofaunal distribution patterns than the eight Arctic Marine Areas (AMAs) defined in the CBMP‐Marine Plan (CAFF, 2017; Gill et al., 2011). Total abundances of meiofauna in each ice core were calculated together with proportional contribution of each taxonomic group within the 14 regions, regardless of sampling period. The majority of all samples were taken between March and August with sites on the shelves and nearshore typically sampled in the earlier months and the central Arctic sites sampled in the later months (Table 1).
5. Separating seasonal fluctuations (and researcher bias) from geographic differences in the entire compiled dataset is difficult. We, therefore, present two seasonal datasets, each collected and analyzed by a consistent researcher team and collected within a small area to remove at least part of the geographic variation: The first study was a multiyear study conducted in Barrow Alaska [Figure 2, same locations as under (2)] investigating landfast ice. This ice in the Barrow area typically starts to freeze in November and breaks up by June–July (Mahoney et al., 2007). Essentially, the same geographic locations were sampled on different dates from April 2002 to June 2007. The second study included the 6‐month long Norwegian young sea ice (N‐ICE 2015) study (Granskog et al., 2016) conducted in the Arctic Ocean north of Svalbard (Figure 2). This study represented mostly first‐year and second‐year pack ice, with modal thicknesses of 1.2 and 1.4 m, respectively (Assmy et al., 2017). The actual geographic sampling location varied with ice drift, and the ice floe sampled changed three times because of relocation of the research vessel (Assmy et al., 2017). The temporal resolutions in these two datasets were irregular and variable with intervals varying from approximately a week to a month. Minimum, maximum, and mean abundance values were calculated for dominant groups, specifically meroplankton (namely polychaete juveniles) as well as Nematoda in landfast ice, and nauplii and Copepoda in pack ice.

### 2.2 Taxonomic grouping of meiofauna

Most studies reported sea ice meiofauna using coarse taxonomic groupings as few taxonomic experts have studied these groups in sea ice, and several taxa do not preserve well with conventional preservatives. Consequently, meiofauna were grouped as follows, using the current taxonomic classification in the World Register of Marine Species (www.marinespecies.org): Chromista including unicellular organisms such as Ciliophora, Foraminifera, and Radiozoa; flatworms including Acoela and Platyhelminthes^1^ (Endnotes); Rotifera; Nematoda; meroplankton^2^ (Endnotes), referring to the usually planktonic larvae and juveniles of benthic organisms, predominantly Polychaeta; Copepoda including Calanoida, Harpacticoida, and Cyclopoida; nauplii including both copepod and noncopepod larvae; Amphipoda; and “others” (including Cnidaria, Ctenophora, Acari, Ostracoda, Pteropoda, and unidentified organisms; Table 2). As nauplii were not consistently identified to higher taxonomic resolution, they were reported on as a group, but across all studies, nauplii of Calanoida, Harpacticoida, and Cyclopoida were observed. Chromista and Amphipoda were excluded from further analyses due to many studies not reporting absolute chromist abundances, and amphipods being considered macrofauna and not quantitatively sampled by ice cores (Gradinger & Bluhm, 2009).

**Table 2:** Composition of meiofauna reported in synthesized literature (23 studies). Taxon groups with subgroups indicated by mean abundance (ind./m^2^) and standard error of mean, aggregate percentage (AP) and standard error, frequency of occurrence (FO) (*n* = 167 for Protozoa and 721 for other groups), and number of ice cores (N) containing a specific group of taxa. Chromista and amphipods were excluded from further analyses. Zero abundance and AP indicate values <0.05

Taxa	Abundance (ind./m^2^)	AP (%)	FO (%)	*N*
Chromista^a^,^b^	21980.7 ± 3724.2	78.4 ± 3.2	76.1	127
Ciliophora^a^,^b^	20789.5 ± 3534.1	76.0 ± 3.3	74.9	125
Foraminifera^a^,^b^	21.4 ± 12.7	0.7 ± 0.6	4.2	7
Radiozoa^a^,^b^	1166.0 ± 1148.6	0.6 ± 0.6	2.4	4
Other^a^,^b^	3.7 ± 3.1	1.2 ± 0.8	1.8	3
Flatworms	1918.0 ± 281.2	8.0 ± 0.7	36.1	260
Rotifera	2552.4 ± 307.1	22.4 ± 1.3	55.8	402
Nematoda	4076.8 ± 636.0	17.0 ± 1.0	55.9	403
Meroplankton	1967.4 ± 332.8	11.7 ± 1.0	34.1	246
Polychaeta	1960.7 ± 332.7	11.6 ± 1.0	32.9	237
Gastropoda	3.5 ± 2.0	0.1 ± 0.1	1.1	8
Bivalvia	3.0 ± 2.4	0.0 ± 0.0	0.7	5
Tunicata	0.2 ± 0.1	0.0 ± 0.0	0.6	4
Cirripedia	0.1 ± 0.0	0.0 ± 0.0	0.3	2
Copepoda	1989.7 ± 311.8	12.9 ± 0.8	54.8	395
Harpacticoida	341.2 ± 57.1	4.5 ± 0.5	26.9	194
Cyclopoida	711.0 ± 163.4	3.1 ± 0.4	16.2	117
Calanoida	310.2 ± 82.2	1.8 ± 0.3	10.5	76
Other	627.3 ± 243.3	3.5 ± 0.5	18.6	134
Nauplii	1141.2 ± 241.2	9.4 ± 0.8	41.8	301
Amphipoda^a^	9.5 ± 2.8	0.1 ± 0.0	2.9	21
Others^c^	87.4 ± 24.1	1.2 ± 0.3	11.5	83
Cnidaria^c^	35.9 ± 7.8	0.2 ± 0.1	5.3	38
Ctenophora^c^	2.0 ± 1.3	0.0 ± 0.0	0.4	3
Acari^c^	2.6 ± 1.7	0.0 ± 0.0	0.6	4
Ostracoda^c^	0.6 ± 0.4	0.0 ± 0.0	0.4	3
Pteropoda^c^	0.3 ± 0.3	0.0 ± 0.0	0.1	1
Unidentified^c^	46.0 ± 22.1	0.9 ± 0.3	5.8	42

a Excluded from further analyses.

b Only studies that reported Chromista included.

c Excluded from multivariate analyses.

### 2.3 Data analysis

#### 2.3.1 Abundance metrics

We used three different metrics to describe our datasets: (1) frequency of occurrence (FO), which presents the proportion of ice cores containing one or more specimens of a given taxon. (2) Abundance metrics used in the published sources were converted to individuals (ind.) per square meter of sea ice (per m^2^) using functions described in Appendix S2. Standard errors were used to express uncertainty of the estimate. (3) Abundance values were neither normally distributed nor homoscedastic and were strongly influenced by outliers. To reduce the influence of outliers, aggregate percentages (Martin, Gensch, & Brown, 1946) were used for compositional analyses. Aggregate percentage (AP) illustrates the mean percentage contribution of a taxon to the total abundance of a sample and was calculated as an arithmetic mean of percentages.

#### 2.3.2 Explanatory environmental variables

We used the entire dataset to identify prominent environmental forcing factors that affect ice meiofauna composition and abundance. As coarse approximations of light availability, day length, and solar angle—as a complementary angle of solar zenith angle—were calculated for each ice core using sampling date, location coordinates, and functions provided by the fish methods package (Nelson, 2015) for R statistical programming environment (R Core Team, 2017). As surrogate for ice type (landfast or pack ice), distance from land and water depth was calculated for each ice core using location coordinates, NOAA bathymetric shapefiles (Amante & Eakins, 2009), and the marmap package (Pante & Simon‐Bouhet, 2013) for R. These variables together with data source (Table 1), sampling month, year, and region were used as explanatory variables in further statistical analyses. Median sea ice extents for Figure 2 were obtained from Fetterer, Knowles, Meier, Savoie, and Windnagel (2016).

For the Bering Sea, additional field‐measured indicators of light availability and conditions inside the ice habitat were used. Snow and ice thickness data were based on field measurements with a meter stick and measured core lengths, respectively. As a measure of food availability, chlorophyll *a* concentration in ice cores was used, measured according to Gradinger et al. (2009).

#### 2.3.3 Statistics

On the individual ice core scale, differences in percentage abundance of meiofauna groups were compared, both within and between ice core sections using permutation analyses of variance (permANOVA; Wheeler & Torchiano, 2016) and pairwise two‐sample permutation tests (Mangiafico, 2017). Resulting *p*‐values were adjusted using the method described in Benjamini and Yekutieli (2001) to avoid false‐positive discoveries.

On the local scale, a multivariate ANOVA [MANOVA; adonis in the vegan package (Oksanen et al., 2017)] was conducted on Bray–Curtis dissimilarity matrices (calculated from species abundance matrices) to examine within‐ and between‐station variability.

A correspondence analysis (CA) was run for regional‐scale Bering Sea and pan‐Arctic meiofauna aggregate percentage data (Oksanen et al., 2017). Explanatory variables were fitted to the CA ordinations as regressions using the vector and factor‐fitting algorithm provided by envfit function from the vegan package (Oksanen et al., 2017). Best‐fitting vectors were further used to constrain the CA ordinations in CCA (Constrained Correspondence Analysis). Bivariate regressions were used to validate the relationships between abundance of taxonomic groups and explanatory variables as indicated by the constraining axes for the CCAs on regional and pan‐Arctic datasets.

## 3 RESULTS

### 3.1 Vertical variability within individual ice cores

**Figure 3:**
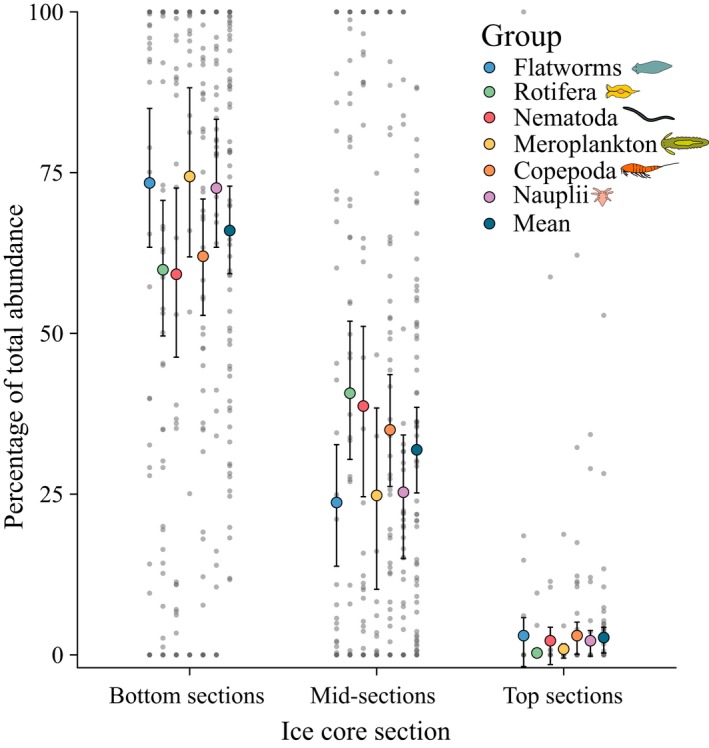
Vertical distribution of sea ice meiofauna in 64 ice cores across the pan‐Arctic domain. The colored dots represent aggregate percentages and indicate average abundance percentage of meiofauna occurring in a respective ice core section. Error bars present bootstrapped 95% confidence intervals for means calculated using underlying data from separate ice cores (gray dots). The three sections (bottom, mid, and top) are explained in material and methods. Only ice cores containing consistent sectioning throughout the core where included in the analysis. See Table 1 for data sources

In the 64 entire cores analyzed, two‐thirds (66.0 ± 3.4%; mean ± standard error of mean [*SE*]; range of 12%–100%) of all meiofauna were found in the bottom 10 cm (Figure 3). Bottom 10‐cm sections showed significantly highest percentage of meiofauna in all taxonomic groups (permANOVA: *df* = 2, 973, *p* < .001; Figure 3), whereas percentage contributions were consistently lowest in top sections (ice–air interface). Contributions of taxonomic groups did not differ significantly within the same core section (permANOVA: *df* = 6, 969, *p* = 1), but there was a trend for higher aggregate percentages of flatworms, meroplankton, and nauplii in the bottom 0‐ to 10‐cm sections and a lower contribution of those groups in midsections.

### 3.2 Small‐scale regional variability in landfast ice

**Figure 4:**
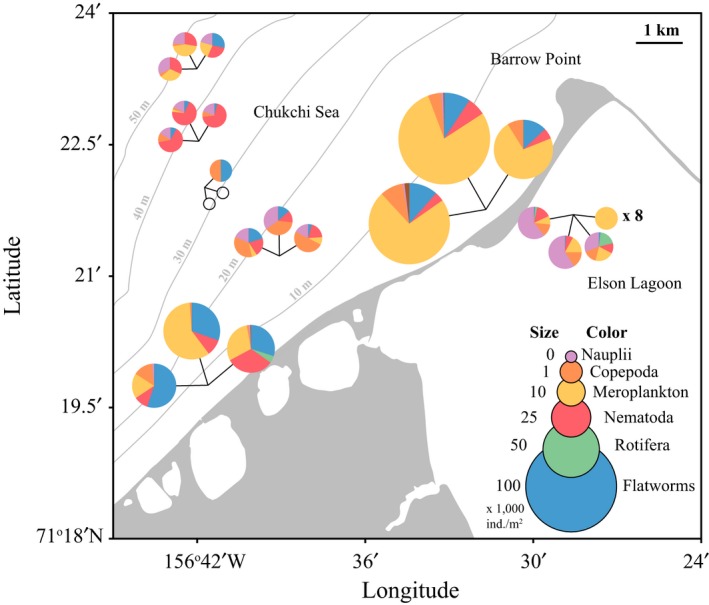
Small‐scale variability in sea ice meiofauna abundance based on bottom 10‐cm sections of ice cores taken close to Barrow, Alaska, within 4 days in April, 2007. Each pie chart represents the lowest 10 cm of an ice core. The size of a chart is related to total meiofauna abundance (1,000 ind./m^2^) within ice core, and pie sections represent different meiofaunal groups. Open circles without color illustrate empty ice cores. At the Elson Lagoon site, eight cores (×8) contained only meroplankton

Variability at the small spatial scale of few kilometers along the coast near Point Barrow, Alaska, was substantial in landfast sea ice in terms of both meiofauna abundance and composition (Figure 4), confirmed by MANOVA where “station” explained 75% of total variation in the dissimilarity matrix (*F*_6,26_ = 10.0, *p* < .001). Specifically, stations at ≤10 m bottom depth had one to two orders of magnitude higher total abundances than those at >10 m depth. Stations ≤10 m also had higher contributions of meroplanktonic stages of benthic taxa, in particular a single species of polychaete, *Scolelepis squamata*, than those >10 m. Stations >10 m were similar to each other in total abundance, but varied in composition. Nauplii were essentially absent at stations ≤10 m, but were prominent at stations >10 m and in some replicates in Elson Lagoon. Copepods (excluding nauplii) consisted almost at equal parts of harpacticoids (55%) and calanoids (45%). Replicates taken at a single station within about 1 m^2^ within 4 days in April 2007 were in five of seven cases broadly similar in composition and abundance. In one of the seven cases (Elson Lagoon, Figure 4), however, some replicates contained only a single taxon, while in yet another one of the seven cases (Chukchi Sea 30 m), two replicates contained no sea ice meiofauna.

### 3.3 Regional variability using the Bering Sea as example

**Figure 5:**
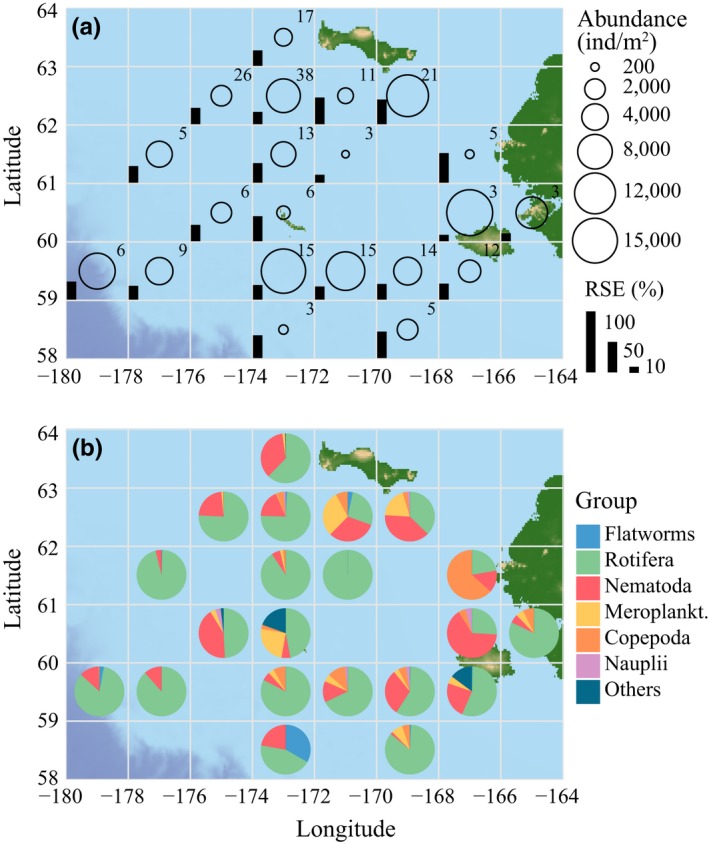
Total abundance (a) and compositional variability (b) in Bering Sea ice meiofauna. A total of 237 ice cores sampled between March 2008 and May 2010 are grouped together within a 1° latitude × 2° longitude grid, and average values are reported. (a) Average total abundance (ind./m^2^, circles) together with number of ice cores in each cell of the grid (number in top right of every cell) and relative standard error of the mean (RSE, bars). If a bar reaches the next latitude, RSE = 100%. If a bar were missing, RSE = 0%. (b) Composition as aggregate percentage (see Methods)

Using the Bering Sea as example, regional‐scale mean meiofauna abundance varied over two orders of magnitude from <200 ind./m^2^ to >15,000 ind./m^2^ within each 1° grid cell with 3–38 cores sampled per grid cell (Figure 5). Ice and snow thickness also varied greatly (ice thickness: 19–152 cm, mean = 66 cm, *SD* = 26 cm; snow depth: 1–32 cm). The dominant taxon in the Bering Sea was Rotifera, which in the majority of grid cells contributed >50% of all individuals. In some grid cells near land (islands, mainland), however, other taxa contributed equal or higher proportions including nematodes, meroplankton, flatworms, and—in a single grid cell—Copepoda (Figure 5). Across all cores, the majority of copepods (excluding nauplii) were harpacticoids (84%).

### 3.4 Pan‐Arctic variability

**Figure 6:**
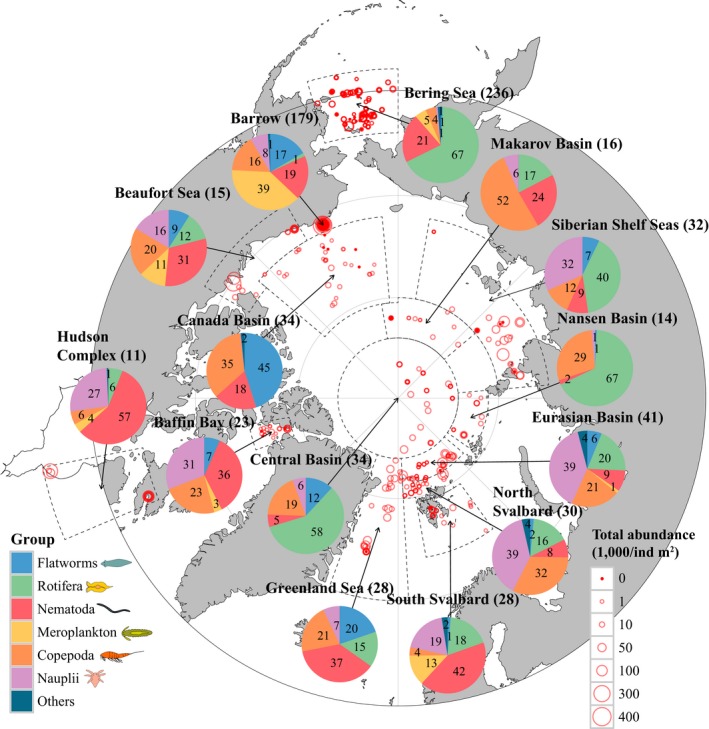
Sea ice meiofauna composition (pie charts) and total abundance (red circles) across the Arctic, compiled from 26 studies between 1979 and 2015 (see Table 1). Scaled circles illustrate the total abundance per individual ice core, while pie charts show average relative contribution by group and region. Number of ice cores for each region is given in parenthesis after region name. Note that studies were conducted at different times of the year, with the majority between March and August (Table 1)

Total integrated meiofauna abundances ranged from 0 to 417,000 ind./m^2^. Maximum abundance values were reached in nearshore locations (landfast ice) and exceeded those of many shelf and basin locations (pack ice locations) by an order of magnitude (Figure 6). Low abundances dominated in basin locations.

**Figure 7:**
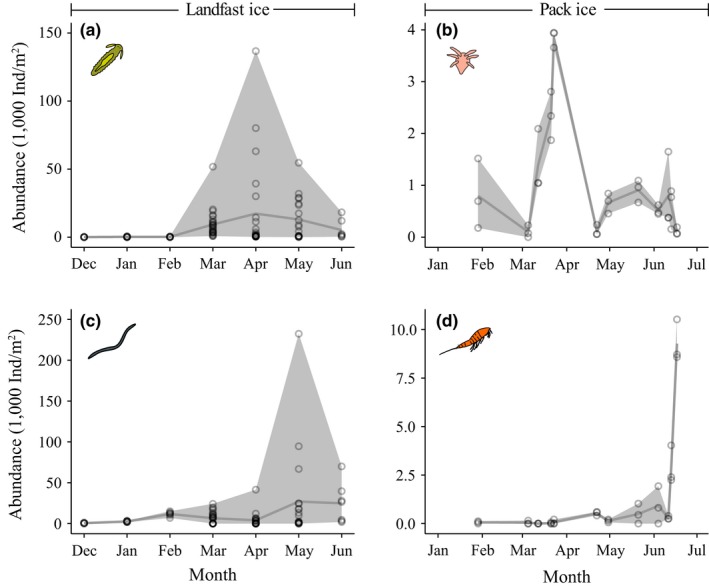
Seasonal abundance (1,000 ind./m^2^) of sea ice meiofauna in landfast ice (Barrow, left panels) and pack ice (north of Svalbard, right panels). (a) Juvenile (meroplanktonic) stages of polychaetes, (b) Nauplii, (c) Nematodes, and (d) Copepods. Circles represent bottom 10‐cm sections of individual ice cores (*n* = 97 for a and c, and *n* = 36 for b and d), shading the extent of minimum as well as maximum values, and gray line indicates mean values

At a coarse taxonomic level, most taxa occurred across the entire Arctic region. The exception was meroplankton, which were mostly confined to coastal locations (Figure 6). Relative proportions of the dominant taxa across the pan‐Arctic varied with region and season (see also Figure 7 for seasonality). While regional comparisons are biased by different sampling months and taxonomic coverage, the aggregated dataset shows that regionally dominant taxa differed among areas (Figure 6). Rotifers dominated or were prominent from the Bering Sea westward across the Siberian shelf seas, and through the Eurasian section of the basins, while nematodes dominated from south Svalbard westward through the Greenland Sea, Baffin Bay, Hudson Complex, and to the Beaufort Sea. Copepods and nauplii combined contributed ≥20% to abundance in any region with ≥60% in the Atlantic advective inflow (north Svalbard, Eurasian Basin). Cnidaria, Ctenophora, Acari, Ostracoda, and Pteropoda contributed little to total abundance in any region (Figure 6, Table 2).

### 3.5 Seasonal variability

Abundances varied seasonally in landfast ice (Barrow, Figure 7a,c) and pack ice (northeast of Svalbard, Figure 7b,d). While absolute abundances were higher at the landfast ice location than in the pack ice area, this difference may also be driven by the different Arctic regions from which these data were obtained. However, the phenology in both datasets was similar in that it showed lowest values in winter and higher, but variable values, during spring and summer. The spring/summer peaks were primarily driven by extremely high abundances in some of the ice cores studied, while other cores still had low abundances (Figure 7). In both datasets, abundance of seasonally ice‐inhabiting juvenile and larval stages peaked earlier (Figure 7a,b, respectively) than those of permanently ice‐inhabiting taxa (Figure 7c,d).

### 3.6 Environmental influences

**Figure 8:**
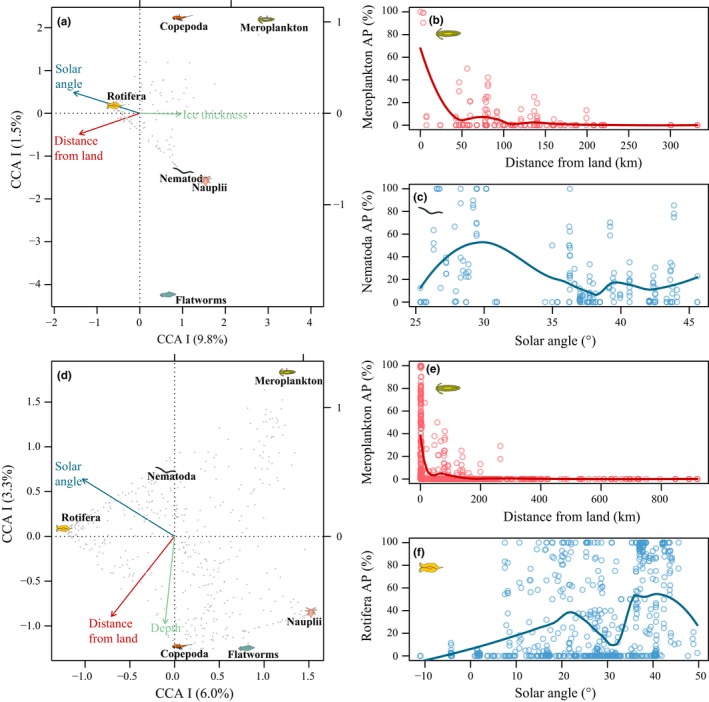
Relationship of environmental factors and ice meiofaunal communities depicted as results of constrained correspondence analysis (CCA) of regional Bering Sea (a) and pan‐Arctic (d) meiofauna data together with bivariate regressions of strongest (albeit generally week) relationships (b, c, e, f). Gray dots in (a) and (d) represent ice cores, taxa denote group contributions, and arrows indicate constraining environmental axes. See Table 3 for detailed overview of explanatory vector and factor fits. Bivariate plots on the right represent meroplankton aggregate percentage relationship with distance from land (b) and nematode relationship with solar angle (c) for the regional dataset, as well as meroplankton relations with distance from land (e) and rotifer relationship with solar angle from the pan‐Arctic dataset (f). Trend lines are local polynomial regressions

Generally, the explanatory variables demonstrated weak and nonlinear relationships with abundances of taxonomic groups (Figure 8, Table 3). Yet, univariate correlations and correspondence analyses (CA) allowed identification of persistent patterns in the datasets. In the regional (Bering Sea) dataset, explanatory variables fitted on the CA orientation using abundance data documented significant contributions of solar angle, distance from land, day length, and ice thickness to the variability in the entire dataset (Table 3), albeit each with *R* ^2^ values <.2. Month and year in the multiyear study also explained a significant part of the variation. Sea ice chlorophyll *a* concentration, reflecting ice algal biomass, and snow depth interestingly had the lowest predictive power of the variables included. Logarithmic transformation increased the *R* ^2^ value for distance to land and bottom depth. Out of the explanatory variables, day length and solar angle were significantly correlated (*r* = .96, *p* < .01), similarly to bottom depth and distance from land (*r* = .47, *p* <.01) as well as ice thickness and snow depth (*r* = .45, *p* < .01). Interestingly, chlorophyll *a* concentration did not significantly correlate with any of the environmental variables. On a taxonomically resolved level, only meroplankton demonstrated both biologically and statistically meaningful relationships with environmental variables, in that meroplankton showed a negative relationship with distance from land (Figure 8a). Meroplankton tended to occur close (0–150 km) to land in highest abundances with a near‐exponential relationship (Figure 8b). Nematodes tended to be more abundant at low solar angles (Figure 8a,c).

**Table 3:** Explanatory factor and vector fit on correspondence analyses (see Figure 8). Bold font indicates significant fit of a given predictor variable

Type	Predictor variable	Transformation	Regional		Pan‐Arctic	
			*R* ^2^	*p*	*R* ^2^	*p*
Vectors	Solar angle	None	.18	**.001**	.13	**.001**
	Distance from land	None	.16	**.001**	.12	**.001**
	Day length	None	.16	**.001**	.01	**.013**
	Ice thickness	None	.06	**.003**		
	Depth	None	.03	.079	.09	**.001**
	Snow depth	None	.02	.154		
	Chlorophyll *a*	None	.01	.291		
	Distance from land	Log(*x*)	.39	**.001**	.40	**.001**
	Depth	Log(*x*)	.22	**.001**	.28	**.001**
	Ice thickness	Log(*x*)	.06	**.001**		
Factors	Month	None	.11	**.001**	.13	**.001**
	Year	None	.08	**.001**	.38	**.001**
	Reference	None			.47	**.001**
	Region	None			.43	**.001**

In the pan‐Arctic dataset, study, region, and year explained best the variability in the CA orientation (Table 3). Similar to the regional dataset, solar angle and distance were the best‐fitting continuous variables with *R* ^2^ values of .13 and .12, respectively (Table 3). Logarithmic transformation increased the fit and *R* ^2^ value for distance from land (.40) and bottom depth (.28). The vector fitting again indicated that meroplankton tended to occur in higher abundances close to land, whereas copepod abundance increased with distance from land and depth (Figure 8d–f). Similar to the regional dataset, these relationships were exponential rather than linear. Rotifer abundances tended to be higher later in the season (i.e., with high solar angle), whereas nauplii were encountered at higher abundances early in the season (i.e., at low solar angle; Figure 8f).

## 4 DISCUSSION

### 4.1 Spatial variability

Ice meiofauna distribution was variable at all scales considered. This observation is in agreement with many regional studies (Gosselin, Legendre, Therriault, Demers, & Rochet, 1986; Mundy, Barber, & Michel, 2005; Wiktor & Szymelfenig, 2002).

Consistent with studies on ice algal distributions (Gradinger, 2009; Schünemann & Werner, 2005), the majority of ice meiofauna was concentrated in the most commonly sampled bottom 10 cm of an ice core. A substantial proportion of around one‐third of the abundance, however, was found further up inside the ice floe. With the goal of capturing meiofauna comprehensively in sea ice in a standardized sampling and analysis approach, we recommend including the 10–20 or 10–30 cm sections in quantitative studies in the future.

While the difference in spatial distribution patterns was not statistically significant among taxa, rotifers, nematodes, and copepods had higher mean contributions away from the water–ice interface than flatworms, meroplankton, and nauplii. All former three taxa have documented tolerance to extreme conditions in temperature and salinity (Heip, Vincx, & Vranken, 1985), which characterize the ice interior away from the ice–water interface. Both nematodes and rotifers withstand extreme conditions through anhydrobiosis, that is, the ability to enter a state of desiccation that lets the organisms persist through extremes (Rebecchi, Altiero, & Guidetti, 2007). Many species of nematodes and rotifers are tolerant to freezing or can avoid freezing by supercoiling (Pejler, Starkweather, & Nogrady, 1983; Wharton, 1995). Rotifers are able to produce resting eggs that await favorable conditions (Gilbert, 1989), or they can mass reproduce through female parthenogenesis when conditions are favorable (Gilbert, 2016). Harpacticoids are tolerant to high salinities occurring in sea ice (Dahms, Bergmans, & Schminke, 1990) as well as freezing into solid ice for short periods (Damgaard & Davenport, 1994). While young stages of benthic polychaetes and flatworms are less stress‐tolerant than nematodes, rotifers, and harpacticoids, species occurring in nearshore environments are still comparatively better adapted to variable and extreme conditions than taxa from less variable environments (Purschke, 1981).

At high taxonomic levels, essentially, the same sea ice meiofauna taxa occurred across the Arctic ice cover. These are predominantly taxa that also occur in the sediment interstitial and include nematodes, rotifers, harpacticoid copepods, and flatworms. As in sea ice, nematodes often dominate benthic meiofauna in abundance globally (Heip et al., 1985) as well as in the Arctic (Vanreusel et al., 2000). Similarly, harpacticoid copepods are often second‐most abundant after nematodes in sediments globally and in the Arctic (Vanreusel et al., 2000). Rotifers, in contrast, are typically not numerically dominant in sediments of the global ocean or the Arctic (Vanreusel et al., 2000), although they can be abundant and species rich in freshwater environments (Fontaneto & Ricci, 2006; Schmid‐Araya, 1998). Perhaps the conspicuous prevalence of rotifers in areas of large river inflow (Figure 6) is related to this fact. As pointed out above, these dominant taxa have a high stress tolerance in common and have mechanisms allowing them to endure environmental conditions commonly occurring in sea ice.

### 4.2 Seasonal phenology

Biological communities in the sea ice system exhibit substantial seasonality linked to the annual cycle in both sea ice formation and light, with strong influence from changes in snow cover (Leu et al., 2015). Few studies actually cover full seasonal cycles; to our knowledge, the two examples given here along with one study on harpacticoid copepods (Kern & Carey, 1983) represent the only ice meiofauna seasonal studies in the Arctic so far. Our analysis of seasonal patterns showed distinct peaks in spring and summer for multiple taxa, but also high variability during these peak times. Seasonal peaks occurred in both ice types considered, although the abundance varied. Nearshore landfast ice typically harbors the highest ice meiofauna spring peaks (>200,000 ind./m^2^; Gradinger et al., 2009; Nozais et al., 2001), followed by shelf pack ice (Gradinger et al., 2009; Marquardt et al., 2011), with the lowest abundances in offshore pack ice and ice pressure ridges (<10,000 ind./m^2^; Friedrich, 1997; Gradinger, Meiners, Plumley, Zhang, & Bluhm, 2005; Gradinger et al., 2010; Schünemann & Werner, 2005). Springtime peaks reflect increased food availability and the onset of the reproductive season.

Different taxonomic groups peak in abundance at different times, a pattern that is related to the trophic and reproductive ecologies of each taxon (examples in Figure 7). Algal grazers peak in abundance in the spring at the onset of the ice algal bloom (Gradinger, 2009), which progresses temporally with increasing latitude through the link to light availability (Leu et al., 2015). Acoel flatworms, platyhelminthes, harpacticoids, and meroplankton, as well as nauplii, are thought to feed on ice algae (Gradinger & Bluhm, 2005; Grainger & Hsiao, 1990) as evidenced by gut content and stable isotope analysis. Although this trophic link seems obvious, ice algal biomass (and snow depth modulating ice algal biomass) lacked explanatory power regarding faunal abundances (Table 3, Figure 8). This might be related to a time lag of faunal compared to algal abundance peaks and the fact that insufficient data on chlorophyll *a*, ice thickness, and snow depth were available to be included in the analysis. Also, nematodes have variable feeding strategies, with the genera common in ice (*Theristus, Monhystera*) thought to feed not only on ice algae but also on bacteria and dissolved organic matter or other nematodes (Grainger & Hsiao, 1990; Tchesunov & Riemann, 1995).

### 4.3 Conclusions and considerations for monitoring

This synthesis documents that, essentially, the same sea ice meiofauna taxa (at the high taxonomic level) occur across the Arctic ice cover, although relative proportions varied widely. It is unclear if the similarity in taxa present is also true at the species level given the general lack of identification to species or even genus, although recent mapping of the sparse species‐level records available suggests that broad species distribution ranges across the Arctic ice cover may indeed be common (Bluhm, Swadling, et al., 2017). Such broad distribution patterns might be expected in pack ice given pan‐Arctic ice drift patterns that can facilitate large‐scale connectivity. Better taxonomic resolution could confirm this notion, for example, through DNA analyses.

Further, we confirm here that patterns in abundance and relative composition of sea ice meiofauna are variable at numerous spatial scales. Regardless of geographic location, the majority of sea ice meiofauna is concentrated in the bottom 10 cm, but a third of all individuals may be found above that layer. Variability at the kilometer scale may be as extreme in abundance and composition as at the regional and pan‐Arctic scales. While environmental factors had overall low explanatory power in our analysis, seasonal variability in ice meiofaunal abundance is directly or indirectly linked to light climate, constrained by solar angle and snow depth, influencing food availability.

The combination of poor taxonomic resolution and large natural variability in abundance and taxonomic composition makes meaningful monitoring of ice meiofauna biota challenging. Even if consistent biological sampling and analyses could be performed to ensure comparability (Gill et al., 2011; Gradinger & Bluhm, 2009), and ice physical and chemical forcing factors were included, what could the data tell us about the changing Arctic? The arrival of completely different phyla in sea ice or a vastly shifting dominance toward previously rare taxa would be detectable. Reductions in biodiversity, indicated by absence of taxonomic groups, have indeed been noted in sea ice studies conducted in the 1990s compared to the 1970s (Melnikov, Kolosova, Welch, & Zhitina, 2002). If additional species knowledge were available, species or community shifts from, for example, large to small species or from fully marine to freshwater species or from being fully endemic or only temporarily present in sea ice would be detectable. Shift in functions such as a change from the currently mostly ice algal‐feeding meiofaunal community (Grainger & Hsiao, 1990) to predatory, detrital, or bacterivore trophic guilds would also be obvious. In conclusion, we suggest to (1) increase taxonomic resolution of sea ice meiofauna, (2) focus on peak abundance sampling where seasonal sampling is impossible, (3) include the bottom 30 cm of ice cores into all analyses, (4) preserve specimens in ethanol to allow DNA analysis, and (5) formulate a trait‐based framework (Bremner, Rogers, & Frid, 2006) that is able to capture ecosystem functioning.

## DATA AVAILABILITY

The data are available in the Norwegian Polar Institute's database (https://doi.org/10.21334/npolar.2017.a5c33604).

## CONFLICT OF INTEREST

None declared.

## AUTHOR CONTRIBUTIONS

BB, HH, and MV wrote the manuscript; BB and HH designed the “experiment”; MV, BB, HH, IAM, RG, IK, and JS collected the data; MV, BB, and RG analyzed the data; BB, HH, IK, RG, MV, JS, and IAM revised the manuscript.

## ENDNOTES

1 Acoela were considered part of Platyhelminthes until the 1990s but are now considered members of the phylum Xenacoelomorpha (Cannon et al., 2016; Ruiz‐Trillo et al., 1999).

2 Merosympagic might be a more appropriate term when such stages are ice‐associated, but we maintain the traditional term meroplankton here.

## References

[ece33797-bib-0001] Amante, C. , & Eakins, B. W. (2009). ETOPO1 1 Arc‐Minute Global Relief Model: Procedures, Data Sources and Analysis. *NOAA Technical Memorandum NESDIS NGDC‐24*. Boulder, Colorado.

[ece33797-bib-0002] Arrigo, K. R. , Matrai, P. A. , & van Dijken, G. L. (2011). Primary productivity in the Arctic Ocean: Impacts of complex optical properties and subsurface chlorophyll maxima on large‐scale estimates. Journal of Geophysical Research, 116, C11022 https://doi.org/10.1029/2011JC007273

[ece33797-bib-0003] Assmy, P. , Fernàndez‐Mèndez, M. , Duarte, P. , Meyer, A. , Randelhoff, A. , Mundy, C. J. , … Granskog, M. A. (2017). Leads in Arctic pack ice enable early phytoplankton blooms below snow‐covered sea ice. Scientific Reports, 7, 40850 https://doi.org/10.1038/srep40850 28102329 10.1038/srep40850 PMC5244362

[ece33797-bib-0004] Barber, D. G. , Hop, H. , Mundy, C. J. , Else, B. , Dmitrenko, I. A. , Tremblay, J. E. , … Rysgaard, S. (2015). Selected physical, biological and biogeochemical implications of a rapidly changing Arctic Marginal Ice Zone. Progress in Oceanography, 139, 122–150. https://doi.org/10.1016/j.pocean.2015.09.003

[ece33797-bib-0005] Benjamini, Y. , & Yekutieli, D. (2001). The control of the false discovery rate in multiple testing under dependency. The Annals of Statistics, 29, 1165–1188.

[ece33797-bib-0006] Bi, H. , Fu, M. , Sun, K. , Liu, Y. , Xu, X. , & Huang, H. (2016). Arctic sea ice thickness changes in terms of sea ice age. Acta Oceanologica Sinica, 35, 1–10. https://doi.org/10.1007/s13131-016-0922-x

[ece33797-bib-0007] Bluhm, B. A. , Hop, H. , Melnikov, I. A. , Poulin, M. , Vihtakari, M. , Collins, E. , … vonQuillfeldt, C. (2017) State of the Arctic Marine Biodiversity Report, pp. 33–61. Conservation of Arctic Flora and Fauna International Secretariat, Akureyri, Iceland.

[ece33797-bib-0008] Bluhm, B. A. , Swadling, K. M. , & Gradinger, R. (2017). Chapter 16: Sea ice as a habitat for macrograzers In Thomas D. N. (Ed.), Sea ice (pp. 394–414). Chichester, UK: John Wiley & Sons Ltd.

[ece33797-bib-0009] Bremner, J. , Rogers, S. , & Frid, C. (2006). Methods for describing ecological functioning of marine benthic assemblages using biological traits analysis (BTA). Ecological Indicators, 6, 609–622. https://doi.org/10.1016/j.ecolind.2005.08.026

[ece33797-bib-0010] CAFF (2017). State of the Arctic Marine Biodiversity Report. Akureyri, Iceland: Conservation of Arctic Flora and Fauna International Secretariat.

[ece33797-bib-0011] Cannon, J. T. , Vellutini, B. C. , Smith, J. , Ronquist, F. , Jondelius, U. , & Hejnol, A. (2016). Xenacoelomorpha is the sister group to Nephrozoa. Nature, 530, 89–93. https://doi.org/10.1038/nature16520 26842059 10.1038/nature16520

[ece33797-bib-0012] Carey, A. G. (1985). Marine ice fauna: Arctic In Horner R. A. (Ed.), Sea ice biota (pp. 173–190). Boca Raton, FL: CRC Press.

[ece33797-bib-0013] Carey, A. G. , & Montagna, P. A. (1982). Arctic sea ice fauna assemblage: First approach to description and source of the underice meiofauna. Marine Ecology Progress Series, 8, 1–8. https://doi.org/10.3354/meps008001

[ece33797-bib-0014] Dahms, H.‐U. , Bergmans, M. , & Schminke, H. K. (1990). Distribution and adaptations of sea ice inhabiting harpacticoida (Crustacea, Copepoda) of the Weddell Sea (Antarctica). Marine Ecology, 11, 207–226. https://doi.org/10.1111/j.1439-0485.1990.tb00240.x

[ece33797-bib-0015] Damgaard, R. M. , & Davenport, J. (1994). Salinity tolerance, salinity preference and temperature tolerance in the high‐shore harpacticoid copepod *Tigriopus brevicornis* . Marine Biology, 118, 443–449. https://doi.org/10.1007/BF00350301

[ece33797-bib-0016] Fetterer, F. , Knowles, K. , Meier, W. , Savoie, M. , & Windnagel, A. K. (2016). Sea ice index, version 2. Boulder, Colorado USA. NSIDC: National Snow and Ice Data Center. Retrieved July 10, 2017 from http://dx.doi.org/10.7265/N5736NV7

[ece33797-bib-0017] Fontaneto, D. , & Ricci, C. (2006). Spatial gradients in species diversity of microscopic animals: The case of bdelloid rotifers at high altitude. Journal of Biogeography, 33, 1305–1313. https://doi.org/10.1111/j.1365-2699.2006.01502.x

[ece33797-bib-0018] Friedrich, C. (1997). Ökologische Untersuchungen zur Fauna des arktischen Meereises. Kiel, Germany: University of Kiel.

[ece33797-bib-0019] Friedrich, C. , & Smet, W. (2000). The rotifer fauna of arctic sea ice from the Barents Sea, Laptev Sea and Greenland Sea. Hydrobiologia, 432, 73–89. https://doi.org/10.1023/A:1004069903507

[ece33797-bib-0020] Gilbert, J. J. (1989). Asexual diapause in the rotifer Synchaeta: Diversified bet‐hedging, energetic cost and age effects. Archive of Hydrobiology Special Issues, Advances in Limnology, 189, 1–9.

[ece33797-bib-0021] Gilbert, J. J. (2016). Resting‐egg hatching and early population development in rotifers: A review and a hypothesis for differences between shallow and deep waters. Hydrobiologia, 796, 1–9.

[ece33797-bib-0022] Gill, M. J. , Crane, K. , Hindrum, R. , Arneberg, P. , Bysveen, I. , Denisenko, N. V. , … Watkins, J. (2011). Arctic marine biodiversity monitoring plan. CAFF Monitoring Series Report No. 3. Akureyri, Iceland.

[ece33797-bib-0023] Golden, K. M. , Ackley, S. F. , & Lytle, V. I. (1998). The percolation phase transition in sea ice. Science, 282, 2238–2241. https://doi.org/10.1126/science.282.5397.2238 9856942 10.1126/science.282.5397.2238

[ece33797-bib-0024] Gosselin, M. , Legendre, L. , Therriault, J.‐C. , Demers, S. , & Rochet, M. (1986). Physical control of the horizontal patchiness of sea‐ice microalgae. Marine Ecology Progress Series, 29, 289–298. https://doi.org/10.3354/meps029289

[ece33797-bib-0025] Gradinger, R. (2009). Sea‐ice algae: Major contributors to primary production and algal biomass in the Chukchi and Beaufort Seas during May/June 2002. Deep Sea Research Part II: Topical Studies in Oceanography, 56, 1201–1212. https://doi.org/10.1016/j.dsr2.2008.10.016

[ece33797-bib-0026] Gradinger, R. R. , & Bluhm, B. A. (2005). Susceptibility of sea ice biota to disturbance in the shallow Beaufort Sea. Phase 1: Biological coupling of sea ice with the pelagic and benthic realms. Fairbanks, AK: University of Alaska Press.

[ece33797-bib-0027] Gradinger, R. R. , & Bluhm, B. A. (2009). Assessment of the abundance and diversity of sea ice biota In Eicken H., Gradinger R. R., Salganek M., Shirasawa K., Perovich D., & Leppäranta M. (Eds.), Handbook of field techniques in sea ice research (a sea ice system services approach) (pp. 283–300). Fairbanks, AK: University of Alaska Press.

[ece33797-bib-0028] Gradinger, R. R. , Bluhm, B. A. , & Iken, K. (2010). Arctic sea‐ice ridges‐Safe heavens for sea‐ice fauna during periods of extreme ice melt? Deep‐Sea Research Part II: Topical Studies in Oceanography, 57, 86–95. https://doi.org/10.1016/j.dsr2.2009.08.008

[ece33797-bib-0029] Gradinger, R. , Friedrich, C. , & Spindler, M. (1999). Abundance, biomass and composition of the sea ice biota of the Greenland Sea pack ice. Deep‐Sea Research Part II: Topical Studies in Oceanography, 46, 1457–1472. https://doi.org/10.1016/S0967-0645(99)00030-2

[ece33797-bib-0030] Gradinger, R. R. , Kaufman, M. R. , & Bluhm, B. A. (2009). Pivotal role of sea ice sediments in the seasonal development of near‐shore Arctic fast ice biota. Marine Ecology Progress Series, 394, 49–63. https://doi.org/10.3354/meps08320

[ece33797-bib-0031] Gradinger, R. R. , Meiners, K. , Plumley, G. , Zhang, Q. , & Bluhm, B. A. (2005). Abundance and composition of the sea‐ice meiofauna in off‐shore pack ice of the Beaufort Gyre in summer 2002 and 2003. Polar Biology, 28, 171–181. https://doi.org/10.1007/s00300-004-0674-5

[ece33797-bib-0032] Grainger, E. H. (1988). The influence of a river plume on the sea‐ice meiofauna in South‐Eastern Hudson Bay. Estuarine, Coastal and Shelf Science, 27, 131–141. https://doi.org/10.1016/0272-7714(88)90086-8

[ece33797-bib-0033] Grainger, E. H. , & Hsiao, S. I. C. (1990). Trophic relationships of the sea ice meiofauna in Frobisher Bay, Arctic Canada. Polar Biology, 10, 283–292.

[ece33797-bib-0034] Grainger, E. H. , Mohammed, A. A. , & Lovrity, J. E. (1985). The sea ice fauna of Frobisher Bay, Arctic Canada. Arctic, 38, 23–30.

[ece33797-bib-0035] Granskog, M. A. , Assmy, P. , Gerland, S. , Spreen, G. , Steen, H. , & Smedsrud, L. H. (2016). Arctic research on thin ice: Consequences of Arctic sea ice loss. Eos, Transactions American Geophysical Union, 97, 22–26.

[ece33797-bib-0036] Heip, C. , Vincx, M. , & Vranken, G. (1985). The ecology of marine nematodes. Oceanography and Marine Biology ‐ an Annual Review, 23, 399–489.

[ece33797-bib-0037] Hunt, G. L. , Drinkwater, K. F. , Arrigo, K. , Berge, J. , Daly, K. L. , Danielson, S. , … Wolf‐Gladrow, D. (2016). Advection in polar and sub‐polar environments: Impacts on high latitude marine ecosystems. Progress in Oceanography, 149, 40–81. https://doi.org/10.1016/j.pocean.2016.10.004

[ece33797-bib-0038] Kern, J. C. , & Carey, A. G. (1983). The faunal assemblage inhabiting seasonal sea ice in the nearshore Arctic Ocean with emphasis on copepods. Marine Ecology Progress Series, 10, 159–167. https://doi.org/10.3354/meps010159

[ece33797-bib-0039] Kiko, R. , Kern, S. , Kramer, M. , & Mütze, H. (2017). Colonization of newly forming Arctic sea ice by meiofauna: A case study for the future Arctic? Polar Biology, 40, 1277–1288. https://doi.org/10.1007/s00300-016-2052-5

[ece33797-bib-0040] Kramer, M. , & Kiko, R. (2011). Brackish meltponds on Arctic sea ice—a new habitat for marine metazoans. Polar Biology, 34, 603–608. https://doi.org/10.1007/s00300-010-0911-z

[ece33797-bib-0041] Krembs, C. , Gradinger, R. , & Spindler, M. (2000). Implications of brine channel geometry and surface area for the interaction of sympagic organisms in Arctic sea ice. Journal of Experimental Marine Biology and Ecology, 243, 55–80. https://doi.org/10.1016/S0022-0981(99)00111-2

[ece33797-bib-0042] Leu, E. , Mundy, C. J. , Assmy, P. , Campbell, K. , Gabrielsen, T. M. , Gosselin, M. , … Gradinger, R. (2015). Arctic spring awakening? Steering principles behind the phenology of vernal ice algal blooms. Progress in Oceanography, 139, 151–170. https://doi.org/10.1016/j.pocean.2015.07.012

[ece33797-bib-0080] Mahoney, A. , Eicken, H. , & Shapiro, L. (2007). How fast is landfast ice? A study of the attachment and detachment of nearshore ice at Barrow, Alaska. Cold Regions Science and Technology, 47, 233–255.

[ece33797-bib-0043] Mangiafico, S. (2017), rcompanion: Functions to support extension education program evaluation. R package version 1.5.7. Retrieved from https://cran.r-project.org/package=rcompanion

[ece33797-bib-0044] Markus, T. , Stroeve, J. C. , & Miller, J. (2009). Recent changes in Arctic sea ice melt onset, freezeup, and melt season length. Journal of Geophysical Research: Oceans, 114, 1–14.

[ece33797-bib-0045] Marquardt, M. , Kramer, M. , Carnat, G. , & Werner, I. (2011). Vertical distribution of sympagic meiofauna in sea ice in the Canadian Beaufort Sea. Polar Biology, 34, 1887–1900. https://doi.org/10.1007/s00300-011-1078-y

[ece33797-bib-0046] Martin, A. , Gensch, R. , & Brown, C. (1946). Alternative methods in upland gamebird food analysis. The Journal of Wildlife Management, 10, 8–12. https://doi.org/10.2307/3795806

[ece33797-bib-0047] Maslanik, J. A. , Fowler, C. , Stroeve, J. , Drobot, S. , Zwally, J. , Yi, D. , & Emery, W. (2007). A younger, thinner Arctic ice cover: Increased potential for rapid, extensive sea‐ice loss. Geophysical Research Letters, 34, L24501 https://doi.org/10.1029/2007GL032043

[ece33797-bib-0048] McConnell, B. , Gradinger, R. , Iken, K. , & Bluhm, B. A. (2012). Growth rates of arctic juvenile *Scolelepis squamata* (Polychaeta: Spionidae) isolated from Chukchi Sea fast ice. Polar Biology, 35, 1487–1494. https://doi.org/10.1007/s00300-012-1187-2

[ece33797-bib-0049] Meier, W. N. , Hovelsrud, G. K. , van Oort, B. E. H. , Key, J. R. , Kovacs, K. M. , Michel, C. , … Reist, J. D. (2014). Arctic sea ice in transformation: A review of recent observed changes and impacts on biology and human activity. Reviews of Geophysics, 52, 185–217. https://doi.org/10.1002/2013RG000431

[ece33797-bib-0050] Melnikov, I. A. , Kolosova, E. G. , Welch, H. E. , & Zhitina, L. S. (2002). Sea ice biological communities and nutrient dynamics in the Canada Basin of the Arctic Ocean. Deep Sea Research Part I: Oceanographic Research Papers, 49, 1623–1649. https://doi.org/10.1016/S0967-0637(02)00042-0

[ece33797-bib-0051] Michel, C. , Nielsen, T. G. , Nozais, C. , & Gosselin, M. (2002). Significance of sedimentation and grazing by ice micro‐ and meiofauna for carbon cycling in annual sea ice (northern Baffin Bay). Aquatic Microbial Ecology, 30, 57–68. https://doi.org/10.3354/ame030057

[ece33797-bib-0052] Moran, S. B. , Lomas, M. W. , Kelly, R. P. , Gradinger, R. , Iken, K. , & Mathis, J. T. (2012). Seasonal succession of net primary productivity, particulate organic carbon export, and autotrophic community composition in the eastern Bering Sea. Deep Sea Research Part II: Topical Studies in Oceanography, 65–70, 84–97. https://doi.org/10.1016/j.dsr2.2012.02.011

[ece33797-bib-0053] Mundy, C. J. , Barber, D. G. , & Michel, C. (2005). Variability of snow and ice thermal, physical and optical properties pertinent to sea ice algae biomass during spring. Journal of Marine Systems, 58, 107–120. https://doi.org/10.1016/j.jmarsys.2005.07.003

[ece33797-bib-0054] Nansen, F. (1906). Protozoa on the ice‐floes of the North Polar Sea. Scientific Results of Norwegian North Polar Expedition, 1893–1896(5), 1–22.

[ece33797-bib-0055] Nelson, G. A. (2015). fishmethods: Fishery Science Methods and Models in R. R package version 1.9‐0. Retrieved from https://cran.r-project.org/package=fishmethods

[ece33797-bib-0056] Nozais, C. , Gosselin, M. , Michel, C. , & Tita, G. (2001). Abundance, biomass, composition and grazing impact of the sea‐ice meiofauna in the North Water, Northern Baffin Bay. Marine Ecology Progress Series, 217, 235–250. https://doi.org/10.3354/meps217235

[ece33797-bib-0057] Oksanen, J. , Blanchet, F. G. , Friendly, M. , Kindt, R. , Legendre, P. , McGlinn, D. , … Wagner, H. (2017). vegan: Community ecology package. R package version 2.4‐4. Retrieved from https://cran.r-project.org/package=vegan

[ece33797-bib-0058] Pante, E. , & Simon‐Bouhet, B. (2013). marmap: A package for importing, plotting and analyzing bathymetric and topographic data in R. PLoS ONE, 8, e73051 https://doi.org/10.1371/journal.pone.0073051 24019892 10.1371/journal.pone.0073051 PMC3760912

[ece33797-bib-0059] Pejler, B. , Starkweather, R. , & Nogrady, T. (1983). Biology of rotifers. 212. http://www.springer.com/gp/book/9789061937654

[ece33797-bib-0060] Perovich, D. , Meier, W. N. , Tschudi, M. , Farrell, S. , Gerland, S. , Hendricks, S. , … Haas, C. (2015). Sea ice. Retrieved from http://www.arctic.noaa.gov/Report-Card

[ece33797-bib-0061] Poulin, M. , Daugbjerg, N. , Gradinger, R. , Ilyash, L. , Ratkova, T. , & von Quillfeldt, C. (2011). The pan‐Arctic biodiversity of marine pelagic and sea‐ice unicellular eukaryotes: A first‐attempt assessment. Marine Biodiversity, 41, 13–28. https://doi.org/10.1007/s12526-010-0058-8

[ece33797-bib-0062] Purschke, G. (1981). Tolerance to freezing and supercooling of interstitial Turbellaria and Polychaeta from a sandy tidal beach of the island of Sylt (North Sea). Marine Biology, 63, 257–267. https://doi.org/10.1007/BF00395995

[ece33797-bib-0063] R Core Team (2017). R: A language and environment for statistical computing. Vienna, Austria: R Foundation for Statistical Computing Retrieved from http://www.r-project.org

[ece33797-bib-0064] Rebecchi, L. , Altiero, T. , & Guidetti, R. (2007). Anhydrobiosis: The extreme limit of desiccation tolerance. Invertebrate Survival Journal, 4, 65–81.

[ece33797-bib-0065] Ruiz‐Trillo, I. , Riutort, M. , Littlewood, D. , Herniou, E. , & Baguñà, J. (1999). Acoel flatworms: Earliest extant bilaterian metazoans, not members of Platyhelminthes. Science, 283, 1919–1923. https://doi.org/10.1126/science.283.5409.1919 10082465 10.1126/science.283.5409.1919

[ece33797-bib-0066] Schmid‐Araya, J. M. (1998). Small‐sized invertebrates in a gravel stream: Community structure and variability of benthic rotifers. Freshwater Biology, 39, 25–39. https://doi.org/10.1046/j.1365-2427.1998.00259.x

[ece33797-bib-0067] Schünemann, H. (2004). Studies on the Arctic pack‐ice habitat and sympagic meiofauna ‐ seasonal and regional variabilities. der Christian‐Albrechts‐Universität zu Kiel http://macau.uni-kiel.de/receive/dissertation_diss_00001205

[ece33797-bib-0068] Schünemann, H. , & Werner, I. (2005). Seasonal variations in distribution patterns of sympagic meiofauna in Arctic pack ice. Marine Biology, 146, 1091–1102. https://doi.org/10.1007/s00227-004-1511-7

[ece33797-bib-0069] Stroeve, J. C. , Serreze, M. C. , Holland, M. M. , Kay, J. E. , Malanik, J. , & Barrett, A. P. (2012). The Arctic's rapidly shrinking sea ice cover: A research synthesis. Climatic Change, 110, 1005–1027. https://doi.org/10.1007/s10584-011-0101-1

[ece33797-bib-0070] Tchesunov, A. V. , & Riemann, F. (1995). Arctic sea ice nematodes (Monhysteroidea), with descriptions of *Cryonema crassum* gen. *n., sp. n. and C. tenue sp. n* . Nematologica, 41, 35–50. https://doi.org/10.1163/003925995X00035

[ece33797-bib-0071] Vanreusel, A. , Clough, L. , Jacobsen, K. , Ambrose, W. G. Jr , Ryheul, V. , Herman, R. , & Vincx, M. (2000). Meiobenthos of the central Arctic Ocean with special emphasis on the nematode community structure. Deep Sea Research Part I: Oceanographic Research Papers, 47, 1855–1856. https://doi.org/10.1016/S0967-0637(00)00007-8

[ece33797-bib-0072] Vaughan, D. G. , Comiso, J. C. , Allison, I. , Carrasco, J. , Kaser, G. , Kwok, R. , … Zhang, T. (2013). Climate change 2013: The physical science basis In Stocker T. F., Qin D., Plattner G.‐K., Tignor M., Allen S. K., Boschung J., Nauels A., Xia Y., Bex V., & Midgley P. M. (Eds.), Contribution of working group I to the fifth assessment report of the intergovernmental panel on climate change (pp. 317–382). Cambridge, UK and New York, NY: Cambridge University Press.

[ece33797-bib-0073] Wharton, D. A. (1995). Cold tolerance strategies in nematodes. Biological Reviews, 70, 161–185. https://doi.org/10.1111/j.1469-185X.1995.tb01442.x 21545388 10.1111/j.1469-185X.1995.tb01442.x

[ece33797-bib-0074] Wheeler, B. , & Torchiano, M. (2016). lmPerm: Permutation tests for linear models. Retrieved from https://cran.r-project.org/package=lmPerm

[ece33797-bib-0075] Wiktor, J. , & Szymelfenig, M. (2002). Patchiness of sympagic algae and meiofauna from the fast ice of North Open Water (NOW) Polynya. Polish Polar Research, 23, 175–184.

