# Supplementary material for: Sea ice meiofauna distribution on local to pan‐Arctic scales
Source: Ecol Evol. 2018 Jan 29;8(4):2350–64. doi: 10.1002/ece3.3797 (PMC5817141; doi:10.1002/ece3.3797)
Supplement: Supplementary file 1 [file ECE3-8-2350-s001.docx]

Ecology and Evolution

Sea ice meiofauna distribution on local to pan-Arctic scales

Supplementary material

# Authors

Bodil A. Bluhm^1,2^, Haakon Hop^1, 3^, Mikko Vihtakari^3^, Rolf Gradinger^1^, Katrin Iken^2^, Igor A. Melnikov^4^, Janne E. Søreide^5^

# Author affiliations

^1^Department of Arctic and Marine Biology, Faculty of Biosciences, Fisheries and Economics, UiT The Arctic University of Norway, N-9037 Tromsø, Norway

^2^University of Alaska Fairbanks, School of Fisheries and Ocean Sciences, Fairbanks, AK 99775-7220, USA

^3^Norwegian Polar Institute, Fram Centre, N-9296 Tromsø, Norway

^4^Shirshov Institute of Oceanology, Russian Academy of Sciences, 117851 Moscow, Russia

^5^The University Centre in Svalbard, PO Box 156, N-9171 Longyearbyen, Norway

# Appendix 1: Unpublished data sources

| Reference | Title/Project/Expedition | Authors | Institute |
| --- | --- | --- | --- |
| Bluhm & Gradinger | Barrow seasonal meiofauna study | Bluhm B.A. & Gradinger R.R | University of Alaska Fairbanks |
| Friedrich | ARKXII 1996 | Friedrich, C. | Alfred Wegener Institute |
| Gradinger, Iken & Bluhm, a | Bering Sea ecosystem survey | Gradinger R.R., Iken K. & Bluhm B.A. | University of Alaska Fairbanks |
| Gradinger, Iken & Bluhm, b | Beaufort Sea cruise | Gradinger R.R., Iken K. & Bluhm B.A. | University of Alaska Fairbanks |
| Hop | N-ICE 2015 | Hop, H. | Norwegian Polar Institute |
| Kramer | Spitsbergen 2009 | Kramer, M | Alfred Wegener Institute |
| Melnikov, a | Arctic-2000 | Melnikov, I. | Shirshov Institute of Oceanology |
| Melnikov, b | APLIS/ICEX | Melnikov, I. | Shirshov Institute of Oceanology |
| Søreide | Van Mijenfjord 2014 | Søreide, J., Gradinger R.R., Marquardt, M. et al. | University Centre in Svalbard |
| Werner & Friedrich | ARKXIII 1997 | Werner, I. & Friedrich, C. | Christian Albrechts University of Kiel |

# Appendix 2: Transformation equations used to calculate ind. m^-2^ abundances

For studies where abundances were given as individuals per ice-core section (ind.), ice-core section ind. m^-2^ values (*y*) were calculated as follows:

$\boldsymbol{y=}\frac{\boldsymbol{1000}\boldsymbol{Chn}}{\boldsymbol{V}}$ (Eq. 1)

Where *C* is the ice-to-water density conversion factor (we used 0.95), *h* the height of the ice-core section in meters, *n* the number of individuals in an ice-core section and *V* the volume of melted ice-core section in liters.

Following equation was used for studies that gave abundances in individuals per liter (ind. L^-1^, y):

$\boldsymbol{y=1000}\boldsymbol{Chn}$ (Eq. 2)

Where parameter abbreviations are similar to Eq. 1

Studies with unit “Ind_theo_” in Table 1 did not record melted ice-core volumes and the ice-core section volume in liter was estimated using volume in for an ideal cylinder:

$$V= 1000\pi hr^{2}$$

Where *h* is height of the ice-core section in meters and *r* the radius in meter.

After transforming abundances to ind. m^-2^ values, the total ice-core abundances (*y_T_*) were calculated by a simple addition:

$$y_{T}= \sum_{i=0}^{N} y_{i}$$

Where *y_i_* is the ind. m^-2^ abundance for ice-core section *i*, and *N* the number of ice-core sections for an ice-core.
